# Informal Caregivers’ Perceptions of Self-Efficacy and Subjective Well-Being When Using Telecare in the Home Environment: A Qualitative Study

**DOI:** 10.3390/geriatrics7050086

**Published:** 2022-08-23

**Authors:** Simona Hvalič-Touzery, Kaja Smole-Orehek, Vesna Dolničar

**Affiliations:** Centre for Social Informatics, Faculty of Social Sciences, University of Ljubljana, 1000 Ljubljana, Slovenia

**Keywords:** assistive technologies, home monitoring devices, subjective well-being, self-efficacy dimensions, informal caregiving for older people, aging society

## Abstract

Background: Self-efficacy (SE) can be used to explain informal caregivers’ ability to cope with the challenges of caregiving. Although SE impacts informal caregivers’ subjective well-being, its effects have not yet been studied from the perspective of telecare use. This study aimed to explore informal caregivers’ perceptions of and associations between SE and subjective well-being when using different telecare functionalities. Methods: A four-month intervention study using a qualitative research design was conducted. In-depth interviews were conducted with 22 informal caregivers of older people who tested one of two telecare devices in their homes. Results: Five task-specific dimensions of caregiver SE were identified that were shaped by telecare use: controlling upsetting thoughts about the care recipient, managing protective vigilance, obtaining support in emergency situations, managing caregiving, work, family life, and responding in a timely manner to changes in the care recipient’s daily routine. These SE dimensions were associated with caregivers’ subjective well-being. Conclusions: Telecare use may contribute to greater caregiver SE and higher subjective well-being. Therefore, future studies should pay more attention to these potential benefits of telecare. Additional dimensions of caregiver SE should be included in existing caregiver SE scales when examining caregiver SE related to telecare use.

## 1. Introduction

According to the EU strategy to support and empower informal caregivers [1], European long-term care (LTC) systems face four major challenges: a growing demand for LTC, the continuous decline in the number of potential professional and informal caregivers (ICs), ensuring the quality of care, and financing LTC. Many European countries, including Slovenia, have reformed their care and support systems toward aging-in-place policies for older persons and the deinstitutionalization of people with disabilities, relying heavily on the support of ICs [2,3,4,5], who are broadly defined as “persons who provide usually unpaid care to someone with a long-term illness, disability or other long-lasting health or care need, outside a professional or formal framework” [1]. ICs form the backbone of care and support systems [4,5]. However, although society expects ICs to provide LTC, existing support measures vary across European countries, and many do not adequately address the needs of ICs [6,7,8], which can lead to financial, emotional, and physical strain for some ICs [9,10]. Furthermore, it is also “unclear how ICs will cope with the increased workload” [5]. Over the last decade, there has been some shift in European policy to address the possibility of using new technologies to support LTC and ICs [7,11,12,13]. It has been recognized that technologies such as telecare “have the potential to support ICs in providing care for people with increasingly demanding needs” [5].

Telecare is a term used for preventive technologies based on information communication technologies [14]. It includes a wide range of telecare devices [15,16] such as personal alarms systems, environmental monitors, mobility-related devices, and reminder systems. These technologies can support ICs in a variety of ways: reducing travel, facilitating long-distance communication, reducing concerns about the safety of older persons in need of care, reducing stress for ICs, etc. [5,17,18]. However, despite the potential of ICs’ use of new technologies, the European Commission has acknowledged the lack of evaluations of technological interventions in LTC for older persons [5,7]. In particular, there is a lack of in-depth understanding of the impact, especially psychological, of the use of new technologies on the ICs of older persons [17]. Even less attention has been paid to studying how telecare use shapes ICs’ self-efficacy (SE)—that is, the confidence ICs have in their ability to cope with the challenges of caregiving.

In addition, as suggested in the literature [19], as we enter an era of increasing technology-enhanced support for ICs, the research community needs to consider a number of important research topics related to technology-enhanced interventions in the context of informal caregiving, such as the facilitators of technology adoption and the methods for evaluating the outcomes and effectiveness of technological solutions.

The aim of this study was therefore threefold: (1) to identify the task-specific dimensions of caregiver SE that may be shaped by telecare use; (2) to identify possible associations between these dimensions and subjective well-being; and (3) to identify possible associations between individual telecare functionalities and caregiver SE.

### 1.1. Background

#### 1.1.1. The Concepts of IC Subjective Well-Being and Self-Efficacy

Based on positive psychology, two approaches can be used to measure psychological well-being: (a) the hedonic perspective focuses on well-being in terms of experiencing positive/negative feelings (affects and emotions) and on the overall assessment of how well life is going; (b) the eudaimonic approach focuses on the importance of a purpose for well-being [20,21]. Our study focuses on the first perspective, often referred to as subjective well-being (SWB) [22,23], and on the extent to which ICs have positive or negative emotional experiences when using telecare, as both are equally important [24]. The psychological resources that influence SWB are also important, especially when individuals face challenges such as balancing work and caregiving. Therefore, following the example of Chudzicka-Czupała and Zalewska-Łunkiewicz [25] we identified perceived SE as a variable that could correspond to important psychological resources for maintaining high levels of SWB.

The concept of SE refers to the personal belief in one’s own ability to successfully accomplish challenging life tasks [26]. Therefore, it plays an important role in a person’s emotions, cognitions, motivations, and behaviors [27]. Because it influences the choice of activities and the motivation to perform or even attempt them, it has been identified in the theory of planned behavior, along with personal beliefs and attitudes, as a key factor influencing behavior change [27,28]. There is some empirical literature about SE and caregiving in which SE is understood as “global, specific to caregiving, or specific to particular caregiving domains/tasks” [29]. Today, the latter conceptualization, which is also used in our study, is preferred, as SE beliefs formulate from specific situations and vary with contextual factors [26,29]. The concept of SE was applied to informal caregiving in the late 1990s, and it has since been found that the theory of SE can be used to explain the variability in the ability of family members to cope with the challenges of caregiving [30]. The caregiver’s SE can be defined as “an internal mediator reflected in individuals’ confidence in their ability to provide self-care and to manage caregiving demands” [31].

The theory of SE suggests that people who possess high SE for performing caregiving tasks are more successful in completing the tasks and report a lower incidence of psychosocial distress and physical illness and lower burnout rates [26]. Researchers also found that higher levels of SE impacted ICs SWB, i.e., reduced negative mood and strain among caregivers [32] and lowered their level of perceived stress [33]. Several scattered dimensions of caregiver SE were found in the literature, none of which were specifically related to the use of telecare. Some of the SE dimensions described were: (i) SE for caregivers’ self-care, (ii) SE for controlling caregivers’ cognitions and disturbing and intrusive thoughts specific to caregiving, (iii) SE for caregivers’ coping, (iv) SE for managing care recipients’ disruptive behaviors, (v) SE for managing care recipients’ (health) symptoms [30,31,34], (vi) IC SE for meeting demands in the caregiving situation [35], and (vii) SE for obtaining respite from family and friends [30].

#### 1.1.2. ICs’ SWB and SE in the Context of Telecare Use

The impact of telecare use on the SWB of ICs, and even more so on that of employed ICs, has been examined in recent literature, although not thoroughly—most studies dealt with this issue only vaguely [17,18,35,36,37,38,39]. Nevertheless, there is some empirical evidence, albeit limited, that the use of telecare can lead to improved peace of mind for ICs, greater reassurance, reduced anxiety and stress, reduced caregiver burden, and thus improved overall caregiver SWB [17,18,19,35,38,40,41]. Numerous authors [26,29,30,33,34,42] have recognized the importance of the caregiver’s SE for preserving their well-being. There is also some evidence of the negative impact of telecare use on ICs’ SWB (e.g., increased anxiety and worry, lack of relief, and increased burden), although these were less pronounced [35,43,44].

A Scottish exploratory study on ICs reported that peace of mind about the welfare and safety of the person they were caring for was the most cited benefit of telecare. In addition, ICs who used telecare felt reassured and therefore less concerned about the safety of the care recipient. Many also reported feeling less stressed and anxious as a result of telecare use [38]. A systematic review found that the existing quantitative evidence tentatively demonstrated the positive effect of telecare on the stress and strain ICs feel due to their caregiving responsibilities, thereby increasing their psychological well-being. However, no evidence was found to suggest benefits in reducing distress or improving quality of life [37]. Nevertheless, an integrative literature review [35] found that one benefit reported in three telecare studies for ICs was a reduction in burden, as measured by standardized scales. Three other studies identified a reduction in the intensity and frequency of stress in employed ICs as a benefit of telecare use. Another recent systematic review that focused on the ICs of people with dementia concluded that all 56 publications included in the analysis reported generally positive experiences of ICs with the use of telecare. It was found that the physical safety of the care recipient is very important to ICs and that telecare provides them with reassurance in this respect. In addition, telecare was found to alleviate worry and burden and improve the overall SWB of ICs [18]. A newer scoping study [17], that focused on the psychological outcomes of telecare use for ICs revealed a lack of research on this topic. Although there were some conflicting findings between studies, six main emotional experiences related to telecare use were identified: peace of mind, reassurance, anxiety, depression, stress, and feeling burdened.

Although the studies presented above are valuable because they examined the effects of telecare use on ICs or ICs’ SE, they could be improved in several ways. First, to our knowledge, caregiver SE and the individual functionalities of telecare services have not been studied from the perspective of telecare use. Second, most of the available resources focused narrowly on the impact of telecare use on ICs’ SWB rather than the reasons for it. Finally, many of the available resources were literature reviews emphasizing the need for empirical studies on this topic.

## 2. Materials and Methods

### 2.1. Study Design and Recruitment

We conducted a four-month intervention study with a qualitative research design [45]. The qualitative methodology played a fundamental role in this study, while quantitative methods were used only for the purpose of collecting objectively measured facts and socio-demographic characteristics. The intervention was carried out in central Slovenia during 2018–2019. A total of 26 dyads were recruited through purposive sampling, including older persons in need of care and their primary ICs. To recruit participants, we involved Zavod Pristan, one of the main providers of home care in 10 Slovenian municipalities (providing care to more than 800 LTC end-users in the community setting) and provider of residential care for 104 residents. This was crucial to gain participants’ trust in the study and to open up emotionally to the researcher who conducted the interviews. Before participants decided to participate in the study, the researcher and the Zavod Pristan care coordinator explained the study process and eCare services to the older people and their informal caregivers in the older people’s homes. Once a dyad agreed to participate, the installation process began and professionals installed the telecare solution to be used in the homes of older people for four months.

In line with the aim of the study, surveys and semi-structured interviews were conducted only with the ICs. Basic health (older person’s frequency of falls, general health status, and the need for help with daily activities), care provision (caregiver’s frequency of care provision and type of care), and socio-demographic data (gender, age, marital status, education, and living situation of and family relationship between the care recipient and IC) were collected in the survey at the beginning of the intervention. Two semi-structured interview guides (the complete interview guide can be found in Supplementary Materilas File S1) were developed for intermediate and final interviews with IC (in the first and fourth month). In the intermediate interview participants were asked about their caregiving situation, their attitude and their first impression of the tested telecare service. In the second interview, they were asked about their use of the tested telecare services, the attitude towards telecare, the perceived usefulness of individual telecare functionalities, the effects of telecare on their daily life, caregiving, and their emotional experiences during telecare use. The interviews lasted 31−91 min, depending on the willingness of participants. The in-depth interviews were audio-recorded and fully transcribed. Personal information was anonymized. Each dyad received a gift voucher (in 100 € value) in appreciation of their time.

### 2.2. Apparatus

The older persons in need of care (i.e., the care recipients) received one of two types of telecare equipment, which were installed in their homes. Their ICs used a telecare mobile app that enabled them to monitor certain activities in the care recipient’s home and receive notifications. Both tested services had a protection unit, motion and door sensors, an emergency pendant, and a mobile app for the ICs, with alarms in the form of push notifications and activity monitoring. In the *first telecare service (TC1)*, used by 14 out of 22 ICs, only the IC is notified in case of an emergency in the form of push notifications when the care recipient presses the emergency pendant. In addition, a service also enables automatic detection of possible user inactivity or unexpected events through detection of various monitoring sensors (motion and contact sensors) and emergency detectors (water leak detector). Once the service is set-up, the push notifications are activated, and the ICs can receive a message on their smartphone or in the app. The *second telecare service (TC2)*, used by 8 out of 22 ICs, also offered a 24/7 call center, a fall detector, and a smoke detector. Assistance at the call center is provided by medical staff. If the call center cannot reach the user, the IC is contacted, or help is organized through the appropriate intervention service (ambulance, fire department, or police). This service enables an immediate call for help by pressing a button on the pendant or the protection unit, or automatic triggering of alarms if the user is unable to make a call for help due to an emergency (e.g., fall, nausea, etc.). The system automatically triggers certain push notifications that are forwarded via the app to the contacts (in our study, the ICs) and the call center.

The tested services were randomly assigned to the participants. The participants were not charged for the use of the equipment or services.

### 2.3. Participants

Due to the restrictive eligibility criteria, purposive sampling was used to identify and select care recipients and their ICs. The eligibility criteria for ICs were that they should (i) be a primary caregiver, (ii) be a family member of an older person in need of care, (iii) be a primary IC providing LTC to an older person for a minimum 4 h a week, (iv) own a smartphone, and (v) have an interest in participating in the study. The exclusion criteria for ICs was poor sight or hearing. The eligibility criteria for the care recipients were that they should (i) have an interest in participating in the study, (ii) be aged 65+, (iii) need help with activities of daily living, and (iv) live alone in their own household. The exclusion criteria for the care recipients were severe cognitive impairment (e.g., dementia) and those requiring intensive care (e.g., immobility after a stroke).

### 2.4. Analysis

We employed a qualitative descriptive approach. The data set was analyzed using content analysis, with a focus on the ICs’ experiences with the telecare system. The analysis was facilitated by the use of the software program Atlas.ti 8. Following a hybrid approach of qualitative methods of content analysis, we incorporated both the data-driven inductive approach and the deductive a priori template of codes approach. The analysis was conducted in several steps, combining theory-driven codes and codes that emerged directly from the data through inductive coding. The data were thus coded and analyzed using both pre-existing codes from previous research as a deductive approach, as well as codes derived from the data themselves as an inductive approach [46,47]. By and large, we followed the standard procedures for qualitative studies, taking inspiration from Saldaña [48], Boyatzis [49], and Braun & Clarke [50]. The analysis process consisted of the first author repeatedly reading the transcripts and performing the coding in several steps, with the first author acting as lead coder. In the first step, all authors read all transcripts without taking notes. In the second reading, the first author drew inspiration from the (albeit scarce) literature on emotional experiences related to telecare use, as discussed in the introduction [17,35,37,38]. Next, deductive coding was performed using the existing classification of emotional experiences in connection to telecare use. A template in the form of codes from a codebook was used as a means of organizing the text for subsequent interpretation [47]. The first author undertook this initial deductive coding based on the first five transcribed interviews, then the codes were discussed with two co-authors to modify and agree on initial codes. The first author then coded the remainder of the transcripts alone. Deductive coding proved to be inadequate, as it did not capture everything the author saw in the data. Furthermore, a partial data analysis revealed the presence of several other telecare use outcomes that elicited different emotional experiences related to telecare use among the ICs of older persons in need of care. Therefore, in the second step, the authors returned to the earlier data and re-coded and refined some codes considering these additional outcomes and some of the inductive codes regarding the emotional experiences related to telecare use (e.g., telecare-induced sense of relief, distrust, and discomfort). In this step, the first author held the role of lead coder, who also involved the other two co-authors in partial cross-checking of the coding and in the process of refining and (re)grouping the codes. Many quotes from the transcripts and emerging ideas were discussed among the three co-authors, and new insights about the data led to the fine-tuning of the codes. This is typical of a qualitative study, in which coding is usually an iterative process that requires the re-validation of previously coded material, as some earlier codes may need to be re-coded, refined, and combined once you have developed your understanding of the data and your code [51]. The coding of all transcripts generated 39 codes on emotional experiences and 46 codes on other telecare use outcomes. These codes were subsequently examined for broader patterns of meaning and in relation to telecare functionalities. In step three, in which the last author also participated, the theory of SE [26] was introduced. Data interpretation involved the independent and consensual interpretation of the patterns in the text. After thorough analysis, positive and negative telecare use outcomes were grouped into five broader dimensions of SE. In addition, the codes on emotional experiences were*—in collaboration between all three authors*—re-examined and grouped into two categories relating to positive and negative emotional experiences regarding telecare use among ICs. In the final step of the analysis, illustrative data (quotations) were extracted [50].

### 2.5. Ethical Review

This study adhered to the Slovenian and international codes of medical ethics, declarations, and conventions and to the General Data Protection Regulation (GDPR). This work was approved by the Slovenian Commission for Medical Ethics on 18 July 2018 (0120-193/2018/15). The participants (i.e., the ICs and the care recipients) were informed about various aspects of the study. Their rights to voluntarily participate and to withdraw from the study at any time without any consequences, as well as their rights to anonymity and confidentiality, were explained to them. Written informed consent was obtained from both the ICs and the care recipients before they were enrolled in the study.

## 3. Results

### 3.1. Characteristics of Study Participants

Of the 26 dyads of care recipients and ICs, 22 completed the study. The ICs ranged in age from 35 to 67 years (M = 53.9, SD = 7.56). Almost two-thirds were female (n = 13). On average, they provided 8.9 h of care per week (SD = 11.90) and had been doing it for an average of 5.3 years (SD = 5.56). Most of the ICs were the care recipient’s children (n = 20), and two were daughters-in-law. The care recipients were, on average, 83 years old (SD = 6.04), ranging from 73 to 92 years old. All but two were female. Six care recipients were in poor health, 10 were in fair health, and 6 were in (very) good health. All but one had fallen at least once in the last 5 years, 14 of whom required medical assistance afterward. Half of them had needed urgent medical help more than once during the preceding 5 years. The Eurofamcare dependency levels [52] were used by the ICs to assess the dependency of the care recipients: five were severely dependent (i.e., unable to perform most daily activities without assistance), eight were moderately dependent (i.e., able to perform some basic daily activities but unable to perform most instrumental daily activities without assistance), and nine were slightly dependent (i.e., able to perform most daily activities but needing assistance with some instrumental daily activities). Sixteen care recipients were unable to perform activities of daily living (e.g., walking, feeding, dressing and grooming, toileting, bathing, and transferring) independently; 13 were only partially able to care for their personal hygiene, and 10 were unable to move outdoors independently. All the care recipients needed some help with instrumental activities of daily living (e.g., household cleaning and maintenance, managing finances and medications, preparing meals, and transportation); 14 of them needed significant help with household cleaning, home maintenance, and financial or administrative tasks (Table 1).

### 3.2. Emergency Interventions and Use of Telecare Solutions during the Intervention

From the beginning of the study, it was agreed that the solution providers would not provide us with quantitative log data from the telecare solutions. We accepted this arrangement, because this was not the focus of the study, as we wanted to capture ICs’ perceptions of and experiences with telecare solutions. We obtained actual information on emergency calls during the intervention study and usage patterns only through the interviews with the ICs. Most of the ICs reported using the app daily, but their usage patterns varied. Most of them viewed the app in the morning to familiarize themselves with the condition of the care recipient’s condition. Some accessed the app a few times a week, while others checked in several times a day to make sure of how the care recipient was doing. *“I was looking at the app all the time, because that worry is just a part of you. You do not stop doing it, and there’s not a day that goes by that you do not worry about it. In the morning, when I get up, the first thing I do is look into it”* (IC14, F, 57). One IC never looked at the app because he was not interested in the app itself but in other functionalities, such as the emergency button and fall detection. During the intervention (Table 2), 5 out of the 22 care recipients had an emergency call sent to either the 24-h call center and/or to the ICs. Moreover, the smoke alarm was activated three times by the same care recipient, and the remaining recorded emergencies were falls, none of which resulted in hospitalization. Sixteen ICs and their care recipients decided to continue using the telecare solution after the intervention ended.

### 3.3. Caregiver Self-Efficacy and Subjective Well-Being

**Figure 1:**
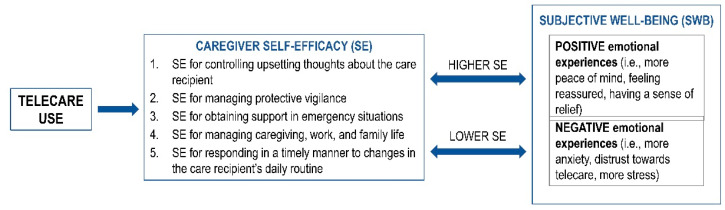
ICs’ SE dimensions regarding telecare.

The data set consisted of 755 pages of transcribed recordings of semi-structured interviews. Based on the stepwise and iterative process of data coding combining deductive and inductive approaches (described in Section 2.4), 22 positive and 24 negative outcomes of telecare use were grouped into five task-specific dimensions of IC SE that are shaped by telecare use. These task-specific SE dimensions evoke eight positive and nine negative emotional experiences related to telecare use (Figure 1, Table 2).

Due to the limited literature on caregivers’ SE dimensions in relation to telecare use, only one dimension that was previously known was identified: SE for controlling upsetting thoughts about the care recipient. The other dimensions that emerged from our findings were not found in any previous studies (Table 3). ICs who possessed higher levels of SE for performing caregiving tasks owing to telecare use reported positive emotional experiences (i.e., more peace of mind, feeling reassured, and having a sense of relief), whereas ICs with lower levels of SE reported negative emotional experiences (i.e., more anxiety, distrust toward telecare, and more stress and strain) (Figure 1). In some general studies on ICs, those with a higher degree of SE for managing caregiving situations experienced positive emotional states, such as less stress [53], more peace of mind [35] and reduced depressive symptoms [42], while lower SE led to negative emotional experiences, such as depression [29,42].

#### 3.3.1. SE for Controlling Upsetting Thoughts about the Care Recipient

First, ICs mentioned that their SE for controlling their upsetting thoughts specific to caregiving was shaped by the use of telecare. This dimension was previously mentioned in the literature and measured as one of three dimensions on the revised scale for caregiving self-efficacy (RSCSE) [30,34,42]. However, this domain of the RSCSE measures upsetting thoughts related to the unpleasant aspects of care provision (i.e., how good life was before caregiving, how much they had to give up due to caregiving, or worries over potential future problems), whereas in our study, the upsetting thoughts were related to ICs’ fear for the safety of their care recipient. Nevertheless, as Steffen et al. [30] noted, “the more efficacious ICs feel about their ability to control distressing thoughts, the more likely they are to do so in a consistent and persistent manner.” Thus, the ultimate achievement is to gain control over upsetting and intrusive thoughts, regardless of their nature.

ICs’ SE for controlling upsetting thoughts about the cared-for person referred to ICs’ sense of control over the negative thoughts regarding the care recipient’s situation. We found several examples of higher IC SE by using telecare. The remote monitoring of the care recipient’s activities allowed caregivers to be sure that the care recipient had arrived home safely, was moving around, and was therefore doing well. Telecare services took a weight off the IC’s mind.

*“Sure, yeah, those safety concerns are a strong pressure. I’ve woken up many times thinking, ‘what if,’ but you’re not going to call the person in the middle of the night, are you? So I’ve thought, who knows if she’s okay or not, especially if she was, let’s say, a little sick the day before. And that was hard for me. Now at least I know if, if something was wrong, she could notify me.”*

(IC14, F, 57)

Several ICs mentioned that being able to rely on telecare reduced their fears when intrusive thoughts about the care recipient’s situation arose, when the care recipient did not answer their phone calls, or when they had an appointment away from home and the caregiver could confirm that they had returned safely.

*“Yes, indeed, that was a concern for me. When I called her and she did not pick up the phone. In such cases, she might have fallen or who knows what worse. Mostly I got the information about her from her neighbour, but much later than I should have. Now it’s really more convenient. If she doesn’t answer, I just check the app to see what room she’s in. I know if something was wrong, she could press the button and I would be notified. That gives me peace of mind. In that sense, I knew she was under control, so to speak.”*

(IC4, F, 45)

Another IC mentioned that, sometimes, the care recipient would hide information from him, which could lead to negative thoughts. Therefore, he felt that telecare gave him a sense of control over the situation and these thoughts:

*“My mother doesn’t want to burden me, so she keeps quiet about some details, she doesn’t even say it, the neighbours do. Maybe that’s why I have an even bigger fantasy, ‘did something happen and she didn’t tell, maybe she just doesn’t want to tell me.’ So you have an extra check, an extra means of having some extra information that allows you to help her, but also to help yourself, so you feel like you’ve done more than just the minimum of the minimum.”*

(IC21, M, 45)

Lower SE for controlling upsetting thoughts was mentioned by only three ICs, who lived within 30 min drive away from the older person. One IC in particular pointed out that false alarms caused her anxiety (e.g., when the app indicated that something might be wrong with the care recipient, especially if they were not responding to calls):

*“As long as the alarm is on, I have that in mind. I’m not going to call her for something unimportant, at seven in the morning.”*

(IC10, M, 66)

Another IC commented about his feelings of worry when he received a push notification that later proved to be a false alarm:

“*These false alarms were a problem for me at first, but later it was ok. But even if you put those notifications aside a little bit, you still think, ‘What if something really is wrong?’”*

(IC22, F, 48)

#### 3.3.2. SE for Managing Protective Vigilance

Another dimension of ICs’ SE that was prominent in all interviews encompassed ICs’ personal sense of control over the unexpected and potentially harmful events for the care recipient, the prompt notification they received, and the general feeling of safety of the care recipient. Several studies [54,55,56] captured ICs’ worries about their care recipients falling or sustaining injuries, which demanded their continuous vigilance, which exhausted them. The literature mentions telecare as a possible substitute for continuous ICs’ vigilance by generating an alarm call or push notification when needed (van Hoof et al., 2013), which was also expressed by ICs in our study:

*“So, you set the device and if she forgets she left something on the stove, then the alarm goes off, so the fear of her burning something is no longer there.”*

(IC15, F, 62)

As one IC put it, higher SE brought them

*“the sense of calm that you say to yourself, look, I see, I call, I know where she is. So there’s definitely a sense of calmness there, which is also why I would like to use it in the future.”*

(IC1, F, 47)

Higher SE led to feelings of reassurance and more peace of mind, which the majority of ICs reported. Furthermore, for some ICs, it also led to a reduction in anxiety and burden, reduced stress, and feelings of relief and joy. Telecare, especially the app’s sensor-based motion detection and its notifications, made it easier for the ICs to ensure the safety of their care recipient. As the ICs reported, telecare enabled them to be aware of what was happening with the care recipients and to avoid endless phone inquiries about them. As all ICs in our study were employed, it was of great value to them that they did not have to also be vigilant while at work—although, as one IC noted, telecare did not reduce his hands-on support:

*“We still have to check on him because, look, if you check on him at six in the morning before you go to work, and in between that woman comes, and it’s five in the afternoon when work is over, and you still have to check on him. But in between, you didn’t worry about it. At work. When you’re at work, you don’t think about him as much. Actually, I don’t think about him at all anymore.”*

(IC12, F, 54)

One IC feared that a potential emergency would not be detected in time and lead to a deterioration of the care recipient’s health (e.g., the older person would get pneumonia) or death. Half of the ICs reported that they felt confident that push notifications in an emergency would allow for a prompt response (from the caregiver or call center), which reassured them that the potential risks to the care recipient were under control, increasing their peace of mind and reducing their anxiety.

One IC, whose father had several cooking accidents that resulted in a fire, felt assured he was now safe from fire-related injuries in his home. Another IC said that times have changed and that she can rely less on the help of the care recipient’s neighbors. She felt that telecare was a kind of substitute for the reduced or lack of help from the neighbors:

*“Now we have this device, which replaces the neighbor as much as possible. Now you look at the phone and you can continue to enjoy the holiday.”*

(IC15, F, 62)

Some ICs also mentioned that it is important for them that the emergency pendant allows the care recipient to call for help themselves in case of an emergency (i.e., a fall, symptoms of a heart attack, or other acute conditions). This gave them a feeling of reassurance and more peace of mind and reduced their fears:

*“In the past, if she fell, you kind of didn’t know she could call someone if she didn’t have a phone nearby. But now she wears an alarm watch on her arm all the time. I’ve been looking for something like this for a while.”*

(IC15, F, 62)

Telecare services did not substitute the constant vigilance of ICs when (i) false alarms were frequent, (ii) the equipment was not fully functional, (iii) the telecare functionality was limited, and (iv) the older person lacked interest in wearing the emergency pendant. This happened to a quarter of the ICs in our study, in particular the ones who lived further away from the care recipient, and it led to negative emotional experiences, such as more anxiety, stress, and less peace of mind:

*“I’ll tell you, this solution almost brought me to the brink of life once when it didn’t work properly. When there was a false alarm.”*

(IC3, M, 56)

Some ICs no longer took push notifications seriously when false alarms occurred repeatedly. For some, these false alarms were so distressing that they turned off one of the most important telecare functionalities—app notifications. Other ICs also mentioned their distrust in motion sensors when the information they saw on the app was incorrect. One IC expressed fear of trusting too much a potentially unreliable device:

*“If something really happened to her, and I didn’t get a text message, I would blame myself, ‘Oh, why did we rely on this device.’”*

(IC2, F, 35)

Some ICs’ lower SE for managing vigilance was also the result of mistrust due to the limited functionality of the telecare solution, fear of having a false sense of security, and doubt that the care recipient would remember to press the alarm button. One IC commented on the limited device functionality:

*“Since there’s still a chance my mother might go somewhere, you can’t solve this with the sensors. Even if I assumed she wasn’t going anywhere right now, I couldn’t really know that she wasn’t going somewhere. So in that respect, it didn’t reassure me.”*

(IC8, F, 54)

#### 3.3.3. SE for Obtaining Support in Emergency Situations

Another dimension that research has shown to be shaped by telecare use is the SE of ICs in emergency situations. This differs from the previous dimension in that it focuses on the ICs’ awareness that they are not alone in the caregiving situation, especially in emergencies, whereas in the previous dimension, the focus was on ensuring the care recipient’s safety. Many studies have reported on the importance of a safety net that enables ICs to continue to care for an care recipient at home [57,58]. A safety net could be provided by various support resources and services, ranging from family and community support (e.g., friends, neighbors) to privately paid assistants and formal services, including new technologies, such as telecare systems [35,36,57,58,59,60].

For the ICs, whose remote monitoring service included support from a call center, it was sufficient to know that someone else was available to help their older relative. For those who did not benefit from call center assistance, it was enough to know that the system would notify them if the alarm was triggered at the care recipient’s home.

Telecare has changed the lives of some participants for the better because they knew that they were no longer on their own. The ICs were relieved, as they no longer felt that everything rested on their shoulders but that someone else out there was watching over their care recipient. As one IC describes the impact of using telecare on her life:

*“You’re not on your own anymore and you don’t have to constantly monitor her, now there’s an app like this to help you do that.”*

(IC13, F, 54)

In addition, this new safety net created by the telecare solution gave half of the ICs a sense of reassurance and more peace of mind and reduced their fears. As one IC put it, it was now twice as likely that somebody would notice an emergency situation and respond to it, as it was before they started using telecare:

*“Now you have someone controlling the situation. A system in the background that works for you. Otherwise, I’d have to take handle it myself.”*

(IC10, M, 66)

Positive emotional experiences (e.g., reassurance) in relation to telecare use were observed among all call center users:

*“Call center is what makes you feel, or let’s say it gives you some time to yourself. You have the feeling that there is someone on duty there and not you.”*

(IC5, M, 55).

However, half of the non-users of the call center said that it would not have given them the feeling of a safety net even if they had used it. The ICs mentioned several issues related to this service: the unfamiliarity of the center with the specific health problems and personal characteristics of the care recipient, the preference of the care recipient to talk to someone close to them rather than with a stranger in an emergency, difficulty of the care recipient to communicate through such a channel (e.g., hearing impairment), and the possible misuse of telecare data. One IC whose mother has communication difficulties expressed reluctance to use a call center:

*“I wouldn’t help myself much with it [referring to a call center] if my mother was alone when she fell. I can assume that she would be able to say something in such a situation, but maybe she wouldn’t be.”*

(IC8, F, 54).

Another IC whose mother has a severe heart condition felt that his mother’s situation was too specific for a call center service to be helpful. He believed that only he could react properly in an emergency:

*“With her illness, everything that happens is serious, and all that is needed is an ambulance and people who are trained to deal with such situations. So in her case, the call center wouldn’t be able to respond that quickly and resolve the situation.”*

(IC6, M, 55)

The lower SE of the ICs was also the result of a mistrust in the functionality of the equipment and the reliability of the telecare service:

*“Yes, that’s what worries me, because last time the alarm went off by itself, I really don’t know why, my mother doesn’t know why either. I don’t know what happened, but then they didn’t call her at all to check on her.”*

(IC14, F, 57)

#### 3.3.4. SE for Managing Caregiving, Work, and Family Life

Informal caregiving is often accompanied by unpredictability and uncontrollability. In particular, ICs find it difficult to strike a balance between domestic life, professional life, and caregiving responsibilities [56,61]. The literature has reported that a conflicting combination of work and informal caregiving is associated with poor well-being, stress, and health problems [56,62]. In addition, evidence shows, albeit to a limited extent, that telecare [63,64,65] can play a significant role in balancing work, caregiving, and domestic life [66].

The vast majority of ICs in this study reported higher SE for managing caregiving, work, and family life when using telecare. They reported being more able to balance their caregiving responsibilities with their work life, other daily activities, and leisure time. In particular, they mentioned better time management and a better work–life balance. Several ICs stated that they found it easier to travel for business or pleasure because they would be alerted via notifications if something went wrong. This reassurance made their work easier and improved their concentration and focus at work:

*“I called her while I was at work. Before noon. Now I don’t. I check the app now and see she’s fine. I spend less time on the calls, to be perfectly honest. I can do a little more at work. Outside of work, I call her just like I used to because we’re used to hearing each other every day.”*

(IC11, F, 67)

Some ICs also said that their confidence in caregiving management had improved, as they were in less of a hurry to visit the care recipient, could focus on other aspects of caregiving, spent less time on some caregiving activities, could more easily provide long-distance caregiving, and made fewer visits or calls just to check on the care recipient which enabled them to find a better balance between caregiving activities and other aspects of their lives:

*“One simply makes other errands more carefree. And you make them easier. And then, of course, everything gets better. It’s a chain reaction.”*

(IC16, M, 48)

The IC providing distant care felt that telecare gave her more free time because fewer visits to the older person were needed:

*“I don’t know what it would be like if I didn’t have this app, I’d probably have to drive to another town several times. It’s pretty stressful for me to drive up and down every day.”*

(IC22, F, 48)

Some ICs mentioned that their lives had become easier and that they could live “a normal life”. One IC also brought up that before using telecare, he often paid a short visit to his mother just to check on her. Now that telecare is providing him with information on his mother’s well-being, the purpose of his visits has changed. Telecare has positively affected his relationship with his mother, as she is more satisfied with him: he can now repair things in the house that he did not have time for before:

*“Now I rarely go to see her just to see her. I go to do something useful, or to keep her company, so she won’t be bored. Not just to check on her. She’s happy with me and even compliments me, which hasn’t happened before.”*

(IC10, M, 66)

ICs recognized the potential of telecare to allow them to participate in leisure activities, social events, and vacations—in other words, to have the opportunity to enjoy a relaxing time:

*“On some occasions you had to rush from a social event immediately, but now you are calm, relaxed, you take your time. For you know that all is well.”*

(IC 15, F, 62).

Since using telecare, only two ICs experienced lower SE in managing caregiving, work, and private life. One IC in particular, who was caring for her mother-in-law, struggled with a sense of moral obligation toward her. Knowing more about the care recipient’s daily routine made the IC’s everyday life more difficult and reduced her confidence in how she managed the caregiving. The IC felt a strong sense of guilt and wondered whether it was her moral duty to call the care recipient more often, pay more visits, or invite her to social gatherings:

*“I have basically lost some peace of mind. I have a bigger problem now because this device has awakened all these things in me, and now I feel some moral responsibility, and before I was not aware of the whole situation. Sometimes I felt bad, but I said to myself, ‘Okay, I’m going to compensate by calling her and giving her more that way.’ Sometimes I was reminded, ‘We should meet with her.’”*

(IC2, F, 35)

#### 3.3.5. SE for Responding in a Timely Manner to Changes in the Care Recipient’s Daily Routine

Powell et al. [67] noted the importance of family member involvement in the timely detection of changes in the health status of their older family members. Although their focus was on the involvement of family members in nursing homes, the same importance can be given to such involvement when caring for their dependent family members at home. Some of the ICs in this study mentioned that the use of telecare made them feel they could identify potential new problems, react in time, and prevent issues before they occurred or became serious—for example, changes in the daily routine of the care recipient. In particular, sensor-based motion detection via the app and app notifications seemed to improve the SE of the ICs in reacting promptly to changes in the care recipient’s daily routine. As one IC mentioned, by gaining a better insight into the care recipient’s daily routine, she and her husband reflected on the future requirements of the informal caregiving they provided and the need to develop a plan to respond in a timely manner to the changing needs of the care recipient:

*“The benefit for me was that I started thinking about all these things. Now we’re much more aware that we need to sit down one day and talk about how we’re going to handle things in the future. It’s better to talk about these things earlier and get the opinion of the loved one you care for when you know she still has sound judgment.”*

(IC2, F, 35)

Some ICs reported that they were reassured that they could respond promptly to some everyday practices that could be potentially harmful to the care recipient. Concern was expressed about not eating or drinking enough and not taking the prescribed medication. One IC, whose mother was severely dependent said:

*“The information about her movement in the apartment also allowed us to communicate with my mother about using the toilet, which is very important for her health. She shouldn’t be accumulating water in her bladder and maybe she wasn’t taking the pill to eliminate water. Now we can see exactly how often she used the toilet. So I would say that the solution has worked for us.”*

(IC6, M, 55)

Familiarity with an care recipient’s daily routine could also provide an IC with important information about the care recipient’s well-being when their daily routine has changed. As one IC pointed out:

*“I basically knew her daily routine. Her daily movements conformed to a pattern with which I became familiar. If something happened, like she went somewhere at the usual time or didn’t get up or something, I knew something wasn’t as it should be and something might have happened to her.”*

(IC5, M, 55)

The use of telecare also led to lower SE in terms of the timely response to changes in the OP’s daily routine, particularly in the case of the aforementioned IC who was looking after her mother-in-law. The additional insights into the care recipient’s daily routine through telecare made the IC feel uncomfortable knowing that the care recipient needed companionship, as the care recipient was alone for two days. This information made the IC feel obligated to pay attention to the care recipient. However, she did not respond to the critical information because her husband—the older person’s son—was reluctant to use telecare, and she felt uncomfortable knowing too much, as she would be invading the care recipient’s privacy. While the potential of telecare services is to provide ICs with information to which they could respond and potentially experience SE, in this example, the caregiver experienced lower SE because of the missed opportunity to respond to potentially negative daily practices. As a result, the IC experienced a negative emotional response from not reacting.

## 4. Discussion

Three important findings concerning ICs’ SE emerge from this analysis: (i) five task-specific dimensions of caregivers’ SE were shaped by the use of telecare (Table 3); (ii) higher levels of ICs’ SE led to positive emotional experiences and thus improved SWB for the ICs; and (iii) an association between the functionalities of telecare services and ICs’ SE was observed.

First, this study provided evidence of a number of caregiver SE dimensions that are shaped by telecare use, only the first of which has been reported previously: (i) controlling upsetting thoughts about the care recipient, (ii) managing protective vigilance, (iii) obtaining support in emergency situations, (iv) managing caregiving, work, and family life, and (v) responding in a timely manner to changes in the care recipient’s daily routine. The use of telecare gave most ICs in our study the belief in their own ability to better control or perform some of the caregiving tasks. In Slovenia, where the needs of ICs are poorly met [2], and no nationwide formal support is available for ICs to relieve them of their care work, solutions such as telecare can be of great importance, as they can provide them with a kind of safety net and the feeling that they are no longer alone in their care situation. Telecare may not relieve them of some of the practical tasks, but it can relieve them in such a way that they do not feel they have to constantly check on the older person they are caring for or worry that they will not respond in a timely manner to a possible negative event, such as a fall or changes in the older care recipient’s life. In addition, the ICs in the study felt better able to balance their family and work lives with caregiving responsibilities. Achieving a high caregiver SE is important because, as Merluzzi et al. [34] found, ICs’ own expectations of their adequacy in caring and dealing with the demands of caregiving play a more important role in the burden and stress than the actual demands of caregiving [34].

Caregiver SE is associated with successful caregiving [30,68], and, as suggested in our study, telecare use may lead to higher levels of caregiver SE. However, it should also be noted that the use of telecare can lead to lower levels of caregiver SE, as was also found in our study, mainly due to the unreliability of the telecare solution, limitations in its functionality, and general IC distrust towards it. At present, studies [18,69] on informal caregivers’ experiences of using assistive devices measure SE with the Generalized Self-efficacy Scale (GSE) [70], the Scale for Caregiving Self-efficacy [71], and the RSCSE [30]. The latter measures three domains of caregiving SE: obtaining respite, responding to disruptive patient behaviors, and controlling upsetting thoughts. Our findings suggest that more appropriate measurement tool(s) should be developed that specifically address caregiver SE when using telecare. Therefore, additional SE dimensions should be added to existing scales when examining SE related to telecare use. The additional dimensions found in our study, along with the dimension measuring technological SE (i.e., belief in one’s ability to successfully accomplish a technologically challenging new task) [72], as some ICs report a lack of technological knowledge and skills [73,74,75], would provide a better and more holistic view of caregivers’ SE when using telecare or another assistive technology.

Second, higher levels of task-specific SE, regardless of which of the five dimensions is considered, improved caregiver SWB as measured by positive and negative emotional experiences, which is consistent with previous findings [32,33]. Our study found that higher levels of SE due to the use of telecare led to reassurance, peace of mind, and a sense of relief among the ICs. Lower levels of IC SE primarily resulted in increased levels of anxiety, distress, and distrust. We also found that lower levels of SE for controlling upsetting thoughts and managing protective vigilance were particularly observed in ICs who lived further away from the care recipient (i.e., up to half an hour’s drive or more) and who perceived telecare as unreliable and limited in its functionalities. They reported negative emotions, such as anxiety, stress, and decreased peace of mind. As Sihto [76] noted, spatial distance and limited opportunities to visit the care recipient are often a source of an IC’s continuous concern, which was exacerbated for some ICs in our study because of the unreliability of the telecare solution used. This study therefore suggests that HMDs need to be fully functional in order to take advantage of telecare opportunities, which can reduce the additional burden of distance and allow families to support their loved ones [77].

Third, in our study, ICs who felt that remote monitoring and the emergency pendant provided a safety net for the care recipients expressed high levels of the observed SE dimensions. After all, ICs tend to give priority to the safety and protection of the care recipient, which can be achieved through a reliable and individualized home remote monitoring service [78]. Some ICs in our study reported how their constant vigilance was reduced by the use of telecare, especially via the remote monitoring of the care recipient’s activities and various detectors that would send a notification to their smartphone in case of emergency. Not only did telecare services give them peace of mind and reassurance regarding the safety of the care recipients, but it also improved their communication with the older people they were caring for. As they received some information from the monitoring, the content of their telephone conversations changed from technical (whether the care recipients had eaten and taken their medication) to richer topics, for which they would normally not have time. This finding is consistent with the results of Mahoney et al. [79,80]. Surprisingly, although all ICs in the present study who used the call center service reported positive emotional experiences in relation to this service, the half of the ICs who did not have access to it did not feel ready to use it for a number of reasons. These results are not aligned with the findings from an earlier Slovenian study [81], in which a care assistance center was perceived as the most important added value of a telecare system or those from a Dutch study in which “the majority of ICs preferred to have the sensor data managed by a professional care center and then be alarmed in case of emergency” [82].

However, looking at our findings from another perspective, teleoperators in such call centers do work in highly controlled settings and under strict practice protocols [83]. Thus, it is “unrealistic to expect call center workers to be as concerned and attentive as a traditional IC providing face-to-face interaction over an extended period of time” [84]. The ICs in our study reported that the activity monitors were particularly beneficial when the care recipient was active and less dependent because they provided ICs with information (e.g., that the care recipient had arrived home safely, was moving around, and was therefore well and safe), while app notifications were particularly beneficial when the care recipient was more dependent and at greater risk of falls or of some other potentially harmful events. Lower caregiver SE and the consequent negative emotional reactions to telecare use were mostly triggered by technical failures and false alarms, although some participants felt less disturbed by these than others [85]. As reported in various studies [86,87,88,89], unreliable and inadequate technology may be harmful for ICs, as well as for the care recipient, in several ways (i.e., the provision of false security, the unsuccessful call in a case of emergency, or the pendant alarm not being worn all the time) and can lead to negative attitudes toward telecare devices, frustration, and refusal to use them regardless of the potential benefits [87,89]. An important factor of acceptance is also a match between the expectations prior to the implementation and the perceived benefits subsequent to telecare use [90]. Some ICs in our study experienced a mismatch, which led to disappointment and the refusal of the telecare service, due to its unreliability in particular. However, many ICs experienced greater SE due to telecare use as a result of the perceived benefits.

Another significant finding that emerged from this study is that telecare acts as a form of support for the majority of ICs and empowers them to provide care. Dinsmore [91] discussed the complexity of changing people’s behavior and stressed the link between improving SE through user experience and empowering people. In the context of informal caregiving, this would mean that ICs learn over time about the impact of telecare use on their lives and the lives of the older people in need and gain confidence in their management of the older people’s care.

This investigation also showed that positive emotional experiences owing to telecare use among ICs are prevalent. These results support the findings of previous sparse studies [17,35,36,39,43,92,93,94] and contribute to new knowledge by showing that reassurance is the most common positive emotional response of ICs to telecare use, ahead of peace of mind and other emotional experiences.

Finally, one additional issue that emerged in our study is related to the question of how the information obtained through the telecare system can be communicated to an older person in need of care. A similar dilemma was pointed out in a study on social workers who support people living in an assisted living accommodation [93]. The author stressed the challenges of wording when following up on the information collected by the remote monitoring service. In particular, they pointed out that they lacked guidelines for follow-up, as they did not want to make the care recipients feel that they were being spied on. Although the relationship between the formal care provider and the care recipient and between the care recipient and their ICs is different, tactfulness in hiding the fact that the passive monitoring system prompted the check-in is a possible strategy. In our study, where the people in a dyad had a close relationship, the observations of passive monitoring were discussed more openly. Therefore, an individualized approach should be used when discussing the information obtained through the telecare service.

### Strengths and Limitations

Our findings should be viewed in light of some methodological limitations. One potential source of bias was the intervention design, which used neither a randomized controlled trial nor a control group. Therefore, we cannot claim that changes in the ICs’ reported SWB resulted from the introduction of telecare services. A second potential source of bias could be the relatively short duration of the intervention. In addition, the scope of this study was limited in terms of the sample, which included only ICs. The third limitation lies in the fact that two co-authors played a much smaller role in the coding and parallel co-coding did not occur, so that calculation of intercoder agreement or interpretive convergence was not possible. A fourth limitation is the relatively small sample size, although a similar or smaller sample size is common in intervention studies using qualitative methods [95] such as ours, due to the of the study’s demanding nature (e.g., installation of telecare in older participants’ homes, longer duration of testing, challenging recruitment process). Another shortcoming was the low incidence of harmful or unexpected events during the test phase; hence, many participants had no real experience with the support and protocols for using the telecare service. Moreover, one of the services offering remote monitoring used devices that were still in the test phase during the intervention study, which can explain the several false alarms that occurred, especially at the beginning of the study. Notwithstanding these limitations, our work offers valuable, in-depth insights into the perspectives of ICs and their emotional experiences during the use of telecare. To our knowledge, this is the first study to examine in detail the impact of telecare use on the SWB of ICs of older persons.

## 5. Conclusions

This study adds to the body of knowledge about the effects of telecare use on various dimensions of ICs’ SE. It also suggests that additional dimensions of IC SE should be included in existing SE scales when examining this construct in the context of telecare use. In particular—based on the qualitative methodological approach that integrated theory-driven codes with data-driven ones—we identified task-specific dimensions of ICs’ SE, which turned out to be important mediators of ICs’ emotional experiences. These findings have significant implications for understanding how SWB is shaped by task-specific dimensions of IC SE, which are important psychological resources for ICs. Furthermore, this study confirms the complex relationships between the functionalities of telecare services and ICs’ emotional experiences with these services. These relationships, together with the impact of other care delivery factors (such as geographic distance), require further empirical and conceptual research.

## Figures and Tables

**Table 1:** The participants’ basic information.

IC ^1^	Age (Years)	Sex	Family Relation	Frequency of Visits	Weekly Hours of Care per Week	Years of Care	Spatial Distance from OP	Subjective Burden of Care	OP’s ^2^ Dependency ^4^	OP’s Overall Health Condition
IC1	47	Female	Daughter	At least once a week	5	5	Within 10 min. drive	Little or no burden	Moderate	Good
IC2	35	Female	Daughter-in-law	At least once a month	5	3	Within walking distance	Little or no burden	Moderate	Fair
IC3	56	Male	Son	At least once a week	3 ^3^	2	Within 30 min. drive	Little or no burden	Slight	Fair
IC4	45	Female	Daughter	(Almost) Every day	4	8	Within 30 min. drive	Moderate burden	Moderate	Poor
IC5	55	Male	Son	(Almost) Every day	5	10	Within 30 min. drive	Moderate burden	Moderate	Good
IC6	55	Male	Son	(Almost) Every day	8	25	Within 10 min. drive	Severe burden	Severe	Fair
IC7	50	Female	Daughter	(Almost) Every day	5	2	Within 30 min. drive	Moderate burden	Moderate	Fair
IC8	54	Female	Daughter	(Almost) Every day	60	<1	Within 30 min. drive	Moderate burden	Severe	Fair
IC9	51	Male	Son	(Almost) Every day	14	7	Within walking distance	Moderate burden	Moderate	Fair
IC10	66	Male	Son	At least once a week	4	<1	Within 30 min. drive	Moderate burden	Moderate	Poor
IC11	67	Female	Daughter	(Almost) Every day	10	1	Within 30 min. drive	Little or no burden	Slight	Very good
IC12	54	Female	Daughter	(Almost) Every day	14	8	Within 10 min. drive	Moderate burden	Severe	Poor
IC13	54	Female	Daughter-in-law	At least once a week	5	1	Within 10 min. drive	Little or no burden	Slight	Fair
IC14	57	Female	Daughter	(Almost) Every day	10	9	Within 30 min. drive	Moderate burden	Severe	Poor
IC15	62	Female	Daughter	At least once a week	5	5	Within 30 min. drive	Moderate burden	Slight	Fair
IC16	48	Male	Son	At least once a week	4	<1	Within 1 h drive	Moderate burden	Slight	Fair
IC17	55	Male	Son	(Almost) Every day	4	2	Within 10 min. drive	Little or no burden	Moderate	Poor
IC18	61	Male	Son	At least once a month	5	5	Within 30 min. drive	Little or no burden	Slight	Very good
IC19	61	Female	Daughter	(Almost) Every day	4	10	Within 10 min. drive	Little or no burden	Slight	Fair
IC20	62	Female	Daughter	(Almost) Every day	12	2	Within 30 min. drive	Moderate burden	Severe	Poor
IC21	45	Male	Son	At least once a week	5	4	Within 30 min. drive	Moderate burden	Slight	Good
IC22	48	Female	Daughter	At least once a week	4	<1	Within 30 min. drive	Little or no burden	Slight	Good

^1^ IC: Informal caregiver; ^2^ OP = Older person in need of care; ^3^ This figure does not include the time spent commuting from the caregiver’s home to the older person’s home, so taking this into account, the caregiver meets the eligibility criteria. ^4^ Dependency levels by Eurofamcare consortium [52]: 1. Severely Dependent—Unable to carry out most activities of daily living, without help (e.g., feeding themselves, or going to the toilet); 2. Moderately Dependent—Able to carry out some basic activities of daily living (for example, bathing, feeding, dressing) but unable without help to carry out most instrumental activities of daily living (e.g., shopping, cooking, housework); 3. Slightly Dependent—Able to carry out most activities of daily living, but requires help with some instrumental activities (e.g., shopping, cooking, housework, etc.); 4. Independent—Able to carry out most activities of daily living, but may need some help occasionally.

**Table 2:** Emergency events during the intervention and telecare use after the intervention.

IC ^1^	Telecare Service ^2^	Emergency Event	Telecare Use after the Intervention
IC1	TC1	No	Yes
IC2	TC1	No	No
IC3	TC1	No	Yes
IC4	TC1	No	Yes
IC5	TC2	No	No
IC6	TC1	No	Yes
IC7	TC1	No	No information
IC8	TC1	No	No
IC9	TC1	Yes (care recipient fall)	Yes
IC10	TC1	No	Yes
IC11	TC1	No	Yes
IC12	TC2	Yes (smoke alarm during cooking)	Yes
IC13	TC2	Yes (care recipient fall)	Yes
IC14	TC2	No	Yes
IC15	TC2	No	Yes
IC16	TC2	No	Yes
IC17	TC2	Yes (care recipient fall)	Yes
IC18	TC2	No	No information
IC19	TC1	No	Yes
IC20	TC1	Yes (care recipient fall)	Yes
IC21	TC1	No	Yes
IC22	TC1	No	No

^1^ IC: Informal caregiver; ^2^ TC1 = Telecare solution consisting of a protection unit, motion and door sensors, an emergency pendant, and a mobile app for the IC, with alarms in the form of push notifications and activity monitoring; TC2 = Telecare solution consisting of a protection unit, motion and door sensors, an emergency pendant, and a mobile app for the IC, with alarms in the form of push notifications and activity monitoring, a 24/7 call center, a fall detector, and a smoke detector.

**Table 3:** Definition of five task-specific dimensions of IC SE shaped by telecare use.

Task-Specific Dimensions of IC SE	Explanation
SE for controlling upsetting thoughts about the care recipient	ICs’ sense of control over negative thoughts (e.g., fear for the safety of their care recipient) regarding the care recipient’s situation.
SE for managing protective vigilance	ICs’ personal sense of control over the unexpected and potentially harmful events for the care recipient (e.g., knowing that they will be notified immediately if something happens).
SE for obtaining support in emergency situations	ICs’ personal belief that they are not alone in the caregiving situation, especially in emergencies.
SE for managing caregiving, work, and family life	ICs’ personal belief in their own ability to balance caregiving, work and family life.
SE for responding in a timely manner to changes in the care recipient’s daily routine	ICs’ personal belief in their own ability to detect changes in the care recipient’s daily routine in a timely manner.

## Data Availability

The data presented in this study are not publicly available due to ethical restrictions.

## Supplementary Materials

The following supporting information can be downloaded at: https://www.mdpi.com/article/10.3390/geriatrics7050086/s1, File S1: Interview guides.
