# Supplementary material for: Informal Caregivers’ Perceptions of Self-Efficacy and Subjective Well-Being When Using Telecare in the Home Environment: A Qualitative Study
Source: Geriatrics (Basel). 2022 Aug 23;7(5):86. doi: 10.3390/geriatrics7050086 (PMC9498651; doi:10.3390/geriatrics7050086)
Supplement: Supplementary file 1 [file geriatrics-07-00086-s001.zip › geriatrics-1835818-supplementary.pdf]

Supplemental Material S1: Interview guides

1. Semi-structured interview guide - INTERMEDIATE

Thank you for agreeing to this conversation and participating in the research. As I have mentioned in the introductory meeting and while describing the General Data Protection Regulation (GDPR), I would like to discuss two key subjects with you today. First, I would like to talk about how you provide care to an older person and the potential challenges you have to face in the caring process. Afterwards, I would like to ask about your experience with the tested telecare service so far, both the positive and negative perspectives.

1.1 Informal care context and its challenges

- What kind of care does your care receiver need? How much care do they need?
- What does the process of providing care look like in your case? How do you organise it? Who is included in providing the care (formal carers, other informal carers)? Are you the primary carer? Does anyone help you? How? With which tasks? How do you divide tasks? What form of care do you perform? What helps you do all this? (including technical solutions, Apps, etc., not exclusively people who provide most care)?
- Do you provide care to any other people? Grandchildren? What kind of care? Do you have any other responsibilities beyond providing care to older person?
- Has anything changed in your life due to providing care to an older person (+/-)? What have those changes been? Can you give an example?
- What types of challenges do you encounter while providing care to an older person? (focus on psychosocial outcomes) (Check up one more time for other issues, for example: are there any major challenges that you can come up with regarding taking care of an older person?)
- What is the biggest challenge that comes with providing care in your opinion (+/-)? How is it reflected in your daily life? How has this challenge impacted your day to day life? How do you deal with it? How do you face this challenge?

1.2 Attitude about new technology

- What is your view on technological innovations such as smartphones, tablets? How skilled are you at using them? Are you able to adapt easily?
- Do you use Apps on your smartphone? Which Apps do you use the most?
- What is your attitude towards smart technology? Do you keep up with the latest developments?

1.3 First impressions about the tested telecare service

- Now I would like to ask you to briefly give me your first impressions from the testing telecare service. What kind of experience was it for you? Please elaborate.
- What was the defining factor that made you decide to participate in the research?
- Did you have any doubts about the tested telecare service before deciding to participate? What were your initial expectations from the tested telecare service? What did you expect from it? What is the most important functionality that the telecare service provides for you? Why?
- Can you briefly describe how you use the telecare service/App (frequency, time, place)?
- What do you see as the biggest advantages of using the tested telecare service? Why? What about the disadvantages? Why?
- Users of 24/7 call centre: Have you used the 24/7 call centre? How many times? What were the reasons?
- What would you point out as the most important/ interesting /surprising (+/-)...? Why?
- Is there anything you would like to add regarding your first impressions from the testing?

2. Semi-structured interview guide - FINAL

Thank you for agreeing to this conversation. Let me quickly remind you of our intermediate interview. We talked about your needs and challenges that you face while taking care of an older person and your first impressions with testing the service. Today, we are going to discuss your experience with the tested telecare service through the entire testing process, the positive and negative psychosocial outcomes, your satisfaction or lack thereof with the tested telecare service and how you experienced it in relation to providing help to an older person.

2.1 Use and experience

- We have already discussed your experience with the tested telecare service in the intermediate interview, now I would like to find out what the experience was like for you in its entire testing period. I would like you to briefly describe your impressions from the start to the end of testing.
- How satisfied are you with the tested service, taking into consideration the entire testing period? Has your level of satisfaction changed since our intermediate interview? What did you like? Could you name something specific that you found especially good about the service?
- I would like to know about the aspects of the telecare service that you did not find satisfactory. What did you dislike? Would you be able to name something that you found particularly troublesome during testing?
- How did you use the service throughout the testing period? Has the use changed since last conversation (place, time, frequency)?
- Was learning to use the tested service easy? Did you find the App easy to use?

2.2 Functionalities of tested telecare services

- We talked about usefulness of tested telecare service in our intermediate interview. Have your views on the usefulness of tested telecare service changed?
- What kind of use do you see for the telecare service? What are its advantages? Disadvantages? What did you find none useful? What would you change? What is missing and should be added? What are some things that you would like to have? What was the most important to you? What was the least important for you? What do you see as the downsides of this service?
- When did you find the telecare service to be the most useful? When was it not?
- What about the functionalities of the service: Do any of the listed functionalities affected your decision regarding whether or not you decide to continue using the tested service?
  The functionalities of tested telecare services are: emergency detectors, sensors, alarm pendant, push notifications, 24/7 call centre
- Which functionality do you find to be the most useful? Which functionality do you find to be the least useful?
- How do the various functionalities of the tested telecare service influence your user experience?
- What would you have liked to have that the tested telecare service did not include?
- Did you encounter any problems with the tested telecare service? How frequently? How did you solve them?

2.3 Changes in caring due to telecare service use

- What has the use of the tested telecare service brought into your everyday life?
- What has changed in your life during the use of the service or because of using it? In what way are the changes reflected?

- What did the tested telecare service change in terms of aforementioned challenges when it comes to caring? Which of the challenges or problems you faced changed, which did not? In what way? If it did not, could the tested telecare service help (partially) resolve any of them, if it included some other solutions? What challenges and what would the solutions be?
- Have you noticed changes due to the use of the tested telecare service in any other aspects of your life (in the workplace, in terms of workplace relationships, in relationships with the older person and other family members, from the point of view of time management, in terms of health and feeling, in terms of physical tiredness, from a financial point of view)? How would you say the tested telecare service has affected these aspects (and which aspects did not affect)?
- What sorts of needs should this telecare service fulfil to help you with your caring? What needs did the service help you fulfil? What did you expect the service to help you with, but it turned out not to? What else would you like to include in the tested telecare service? What would you point out as the most important/interesting? Why?

2.4 Psychosocial outcomes after telecare service use

- We talked about your psychosocial outcomes when using the tested telecare service in the intermediate interview. Now I would like to know how you experienced the use of the tested telecare service from start to finish of testing period. What psychosocial outcomes did you experience while using it (positive and negative)?
- How did you feel while using the tested telecare service?
- Has the tested telecare service helped in reducing your burden in terms of caring? How did it do that? In what way? Why?
- How could the tested telecare service reduce your burden further? What kinds of solutions would it need to provide further peace of mind? Why?
- Did using the tested telecare service reduce or increase your stress levels?
- Are you more or less calm? Are you more or less nervous?
- Did using the tested telecare service increase or decrease your burden in terms of caring for an older person? Did you have any concerns regarding the App?
- Do you find yourself to be less pressured (emotionally, in terms of time)?
- Do you feel that using the tested telecare service lets you better or worse deal with situations related to caring for an older person?
- Do you feel like use of the tested telecare service gives you more or less control over situations related to caring for an older person?
- Do you feel like using the tested telecare service has made you more or less confident regarding caring for an older person? Do you feel more or less confident when caring for an older person?
- While using the tested telecare service, did you feel reassured that the older person was safe?
- Do you feel that the tested telecare service has made it easier or harder for you to adapt to daily activities?
- Do you feel that your life was easier or harder when using the tested telecare service?
- Do you feel that caring is easier or harder when using the tested telecare service?
- Did you or did not you feel depressed? (Only ask if relevant)

2.5 Relationship with new technology after tested service use

- Has using the tested telecare service changed your attitude towards new technology? In what way?
- Would you accept the tested telecare service as something to use daily? Why yes/why not? What are the main factors for this answer? In what case would you?
- Do you intend to use the tested telecare service within one year? (if it is available on the market) Why yes/why not?
- Would you recommend the tested telecare service to anyone? To whom? Why?

- What would you suggest to the developers/providers of the tested telecare service that would improve your informal caring situation? (e.g. video monitoring, call centre, clock, etc.).

2.6 Conclusion

- Is there anything else that you would like to add?
